# Annual vaccine-preventable disease report for New South Wales, Australia, 2014

**DOI:** 10.5365/WPSAR.2016.7.3.006

**Published:** 2017-06-26

**Authors:** Nathan Saul, Robin Gilmour, Paula Spokes

**Affiliations:** a Communicable Disease Branch, NSW Health.

## Abstract

This report provides an epidemiological description of selected vaccine-preventable diseases in New South Wales (NSW), Australia, for 2014 to inform ongoing disease monitoring and control efforts. A trend of increasing pertussis notifications was observed, beginning midway through 2014 with the highest disease rates in the 5–9 year age group. Measles notifications increased to 67 cases in 2014 from 34 cases in 2013. Measles cases were associated with travel-related importations—predominantly from the Philippines—and secondary transmission increased compared to 2013 involving three main disease clusters. Notifications of invasive meningococcal disease continued to decline across the state with meningococcal B remaining the most common serogroup in NSW. Increasing rates of pertussis notifications from mid-2014 may indicate the beginning of an epidemic, ending the period of low transmission observed in 2013 and the first half of 2014. An increase in measles notifications in 2014, including secondary transmission, indicates the continued need for public health actions including robust follow-up and awareness campaigns.

## Introduction

Australia has a national immunization schedule funded by the Commonwealth Government and administered by the states and territories with recommended vaccines listed in the Australian Immunization Handbook. (*1*) States and territories are responsible for public health follow-up and maintaining notification databases on conditions with nationally defined case definitions. (*2*)

Monitoring vaccine-preventable diseases enables identification of high-priority events that require urgent attention and facilitates public health response. Ongoing monitoring also enables identification of high-risk groups, changes in affected groups over time, public health interventions and informing policy and programmes.

New South Wales (NSW) is divided into 15 local health districts (LHDs), each with 12 public health units (PHUs). PHUs have the responsibility to follow up on events of public health significance, including vaccine-preventable diseases. Medical practitioners, hospital general managers and diagnostic laboratories are required to notify certain conditions under the State’s public health legislation. (*3*) These notifications are reviewed by PHU surveillance officers and, if consistent with the case definition, are entered into the NSW Notifiable Conditions Information Management System (NCIMS).

This report describes the notifications for diphtheria, invasive *Haemophilus influenzae* type b disease, measles, mumps, invasive meningococcal disease (IMD), pertussis, invasive pneumococcal disease (IPD), rubella and tetanus in NSW for 2014.

## Methods

Cases were notified if they met the nationally agreed confirmed or probable case definition (*2*) and had a condition onset date in 2014. Information on each of these notifications was collected as part of standard PHU case follow-up as described in the NSW and national control guidelines (*4*) and entered into NCIMS as per reporting legislative requirements.

Crude annual disease incidence rates were calculated by year of notification and by age group for 2014 using HealthStats NSW (*5*) population estimates (modified from Australian Bureau of Statistics data).

Case counts and rates were analysed by age, sex, vaccination status (verified through the Australian Childhood Immunization Register, general practitioners or health care records where possible) and geographic residence where information was available.

### Ethics

This work involved the use of NSW disease notification data and was collected as part of standard public health action; as such, no ethics approval was required.

## Results

**Table 1** shows the total number of notifications and rate per 100 000 each year since 1991, **Table 2** shows notifications and rates by age group, and **Table 3** shows notifications and rates by LHD.

**Table 1:** Number and rate per 100 000 of case notifications by year of onset for selected vaccine-preventable diseases, New South Wales, Australia, 1991 to 2014

Year	*Haemophilus influenzae* type b (invasive)		Measles		Meningococcal disease (invasive)		Mumps		Pertussis		Pneumococcal disease (invasive)*		Rubella		Tetanus	
	n	Rate	n	Rate	n	Rate	n	Rate	n	Rate	n	Rate	n	Rate	n	Rate
2014	6	0.1	67	0.9	36	0.5	79	1.1	3131	41.7	516	6.9	10	0.1	1	0.0
2013	9	0.1	34	0.5	46	0.6	91	1.2	2342	31.6	472	6.4	12	0.2	2	0.0
2012	2	0.0	172	2.4	65	0.9	105	1.4	5843	80.0	581	8.0	10	0.1	1	0.0
2011	4	0.1	90	1.3	71	1.0	68	0.9	13 197	182.8	530	7.3	17	0.2	1	0.0
2010	6	0.1	26	0.4	73	1.0	40	0.6	9344	130.8	497	7.0	13	0.2	1	0.0
2009	6	0.1	19	0.3	92	1.3	40	0.6	12 549	177.9	476	6.8	7	0.1	2	0.0
2008	8	0.1	39	0.6	80	1.2	77	1.1	8754	126.1	546	7.9	17	0.2	1	0.0
2007	7	0.1	4	0.1	109	1.6	323	4.7	2096	30.7	519	7.6	8	0.1	2	0.0
2006	11	0.2	60	0.9	101	1.5	155	2.3	4910	72.8	562	8.3	37	0.6	2	0.0
2005	7	0.1	5	0.1	137	2.1	111	1.7	5796	86.6	642	9.6	10	0.2	1	0.0
2004	5	0.1	12	0.2	146	2.2	65	1.0	3564	53.6	903	13.6	17	0.3	1	0.0
2003	6	0.1	18	0.3	197	3.0	36	0.5	2769	41.8	801	12.1	23	0.4	1	0.0
2002	10	0.2	8	0.1	212	3.2	29	0.4	2014	30.6	881	13.4	35	0.5	0	0.0
2001	7	0.1	31	0.5	232	3.6	28	0.4	4436	67.9	444	6.8	58	0.9	0	0.0
2000	8	0.1	35	0.5	248	3.8	91	1.4	3693	56.9	ID	ID	191	2.9	3	0.1
1999	13	0.2	34	0.5	217	3.4	33	0.5	1413	22.0	NN	NN	45	0.7	1	0.0
1998	11	0.2	119	1.9	185	2.9	38	0.6	2306	36.4	NN	NN	78	1.2	3	0.1
1997	17	0.3	272	4.3	218	3.5	30	0.5	4243	67.6	NN	NN	153	2.4	3	0.1
1996	13	0.2	191	3.1	161	2.6	27	0.4	1154	18.6	NN	NN	631	10.2	1	0.0
1995	29	0.5	596	9.7	113	1.8	14	0.2	1368	22.3	NN	NN	2374	38.8	0	0.0
1994	61	1.0	1483	24.5	142	2.3	11	0.2	1405	23.2	NN	NN	229	3.8	4	0.1
1993	124	2.1	2345	39.1	153	2.6	13	0.2	1533	25.5	NN	NN	1184	19.7	5	0.1
1992	217	3.6	804	13.5	121	2.0	23	0.4	218	3.7	NN	NN	323	5.4	2	0.0
1991	212	3.6	494	8.4	128	2.2	8	0.1	49	0.8	NN	NN	59	1.0	5	0.1

* Invasive pneumococcal disease was not notifiable (NN) until 2000; in 2000 there were incomplete data (ID).

**Table 2:** Number and rate per 100 000 population of case notifications by age group for selected vaccine-preventable diseases in New South Wales, Australia, 2014

Age group (years)	*Haemophilus influenzae* type b (invasive)		Measles		Meningococcal disease (invasive)		Mumps		Pertussis		Pneumococcal disease (invasive)		Rubella		Tetanus	
	n	Rate	n	Rate	n	Rate	n	Rate	n	Rate	n	Rate	n	Rate	n	Rate
0–4	3	0.6	12	2.4	14	2.8	7	1.4	400	80.8	69	13.9	0	0	0	0
5–9	0	0.0	4	0.9	0	0	8	1.7	636	134.6	11	2.3	1	0.2	0	0
10–14	0	0.0	6	1.3	3	0.7	3	0.7	518	115.2	5	1.1	0	0	0	0
15–19	0	0.0	15	3.2	3	0.7	9	1.9	122	26.3	9	1.9	0	0	0	0
20–24	0	0.0	11	2.2	1	0.2	7	1.4	86	17.0	6	1.2	1	0.2	0	0
25–29	0	0.0	8	1.5	0	0.0	1	0.2	82	15.2	12	2.2	3	0.6	0	0
30–34	1	0.2	6	1.1	2	0.4	10	1.8	128	23.6	11	2.0	0	0	1	0.2
35–39	0	0.0	4	0.8	0	0.0	9	1.8	128	25.6	20	4.0	2	0.4	0	0
40–44	0	0.0	1	0.2	1	0.2	2	0.4	175	33.3	18	3.4	1	0.2	0	0
45–49	0	0.0	0	0	0	0	3	0.6	145	29.9	38	7.8	2	0.4	0	0
50–54	0	0.0	0	0	1	0.2	4	0.8	125	25.0	30	6.0	0	0	0	0
55–59	0	0.0	0	0	1	0.2	4	0.9	122	26.4	41	8.9	0	0	0	0
60–64	1	0.2	0	0	1	0.2	4	1.0	127	31.0	46	11.2	0	0	0	0
65–69	1	0.3	0	0	3	0.8	3	0.8	103	27.9	49	13.3	0	0	0	0
70–74	0	0.0	0	0	1	0.4	2	0.7	80	29.5	34	12.5	0	0	0	0
75–79	0	0.0	0	0	2	1.0	2	1.0	68	33.0	31	15.0	0	0	0	0
80–84	0	0.0	0	0	0	0	1	0.7	48	31.2	32	20.8	0	0	0	0
85+	0	0.0	0	0	3	1.9	0	0	37	23.1	54	33.8	0	0	0	0

**Table 3:** Number and rate per 100 000 population of case notifications by local health district^#^ for selected vaccine-preventable diseases in New South Wales, Australia, 2014

Local Health District	*Haemophilus influenzae* type b		Measles		Meningococcal disease		Mumps		Pertussis		Pneumococcal disease (invasive)		Rubella		Tetanus	
	n	Rate	n	Rate	n	Rate	n	Rate	n	Rate	n	Rate	n	Rate	n	Rate
Central Coast*	0	0	11	3.3	3	0.9	1	0.3	24	7.3	21	6.3	3	0.9	0	0
Far West*	0	0	0	0	0	0	0	0	23	74.6	6	19.5	0	0	0	0
Hunter New England	1	0.1	1	0.1	11	1.2	3	0.3	441	48.8	79	8.8	0	0	0	0
Illawarra Shoalhaven*	0	0	1	0.3	1	0.3	10	2.5	164	41.5	20	5.1	0	0	0	0
Mid North Coast*	1	0.5	0	0	0	0	1	0.5	27	12.7	18	8.5	0	0	0	0
Murrumbidgee*	0	0	0	0	2	0.8	3	1.3	233	97.5	20	8.4	0	0	0	0
Nepean Blue Mountains*	1	0.3	1	0.3	2	0.6	4	1.1	177	49.0	25	6.9	0	0	0	0
Northern NSW*	1	0.3	2	0.7	1	0.3	1	0.3	61	20.7	14	4.8	0	0	1	0.3
Northern Sydney	0	0	8	0.9	3	0.3	12	1.4	427	48.0	44	5.0	1	0.1	0	0
South Eastern Sydney	0	0	12	1.4	4	0.5	14	1.6	530	60.1	56	6.4	2	0.2	0	0
South Western Sydney	0	0	5	0.5	4	0.4	9	1.0	149	16.1	69	7.5	1	0.1	0	0
Southern NSW*	0	0	0	0	1	0.5	1	0.5	147	72.6	14	6.9	0	0	0	0
Sydney	0	0	12	2.0	0	0	8	1.3	144	23.5	32	5.2	1	0.2	0	0
Western NSW*	0	0	4	1.4	2	0.7	1	0.4	149	53.8	27	9.7	0	0	0	0
Western Sydney	2	0.2	10	1.1	1	0.1	11	1.2	427	47.1	66	7.3	2	0.2	0	0

Note: Some (*) LHDs have small populations with fewer than 500 000 people and a small number of cases. As such, the rates per 100 000 can be unstable.

^#^ Immunization coverage data by LHD are available in annual immunization coverage reports. (*6*)

### Diphtheria

No cases of diphtheria were notified in NSW in 2014. However, one case of cutaneous diphtheria, which is not notifiable in NSW, was reported in a male in his 60s. Public health investigation found that the infection was acquired in Indonesia.

### Invasive *Haemophilus influenzae* type b disease

Six cases of invasive *Haemophilus influenzae* type b disease were notified in 2014 (0.1 cases per 100 000 population). This rate has been consistent over the past 10 years, indicating a low level of infections.

Three of the six cases were less than 5 years of age; of these, one was fully vaccinated for age, and one was partially vaccinated for age. All other cases occurred in unvaccinated individuals. One of the six cases was female.

### Measles

Measles notifications increased from 34 cases in 2013 to 67 cases in 2014. Secondary transmission occurred and was associated with three main disease clusters following the initial importation. Of the 67 cases, 28 were acquired outside Australia and two additional cases outside NSW. Most importations were from the Philippines (12 cases), Viet Nam (seven cases) and Indonesia (five cases). Females comprised 45% of cases in 2014.

Of the 67 cases, 49 occurred in individuals who were either not vaccinated or did not know their vaccination status. Of the other 18 cases, 16 were vaccinated (five were recorded as receiving one dose, five with two doses, six with unknown number of doses) and two had no information collected on vaccination status. Thirty-four of the cases were genotyped: 21 were genotype B3, 10 genotype D8, two genotype D9 and one genotype G3.

### Mumps

Mumps notifications slightly decreased to 79 cases in 2014 compared to 91 in 2013. The highest rates were reported in adolescents and young adults. Females comprised 54% of cases. Mumps cases are not routinely followed up by PHUs in NSW.

### Invasive meningococcal disease

Thirty-six cases of IMD were notified in 2014, down from 46 cases in 2013. Two deaths occurred among these cases: one was in the 0–4 year age group (serogroup B) and one was in the over 85 year age group (serogroup Y). The highest rate of disease was in the 0–4 year age group (2.8 cases per 100 000 population; 14 cases) followed by the over 85 year age group (1.9 cases per 100 000 population; three cases). Females comprised 44% of cases in 2014.

All of the cases notified in 2014 had a serogroup identified. Serogroup B was the most common, with 22 cases accounting for 61% of notified IMD in NSW. Serogroup Y and serogroup W135 each accounted for 19.4% of cases (seven cases each).

### Pertussis

**Fig. 1:**
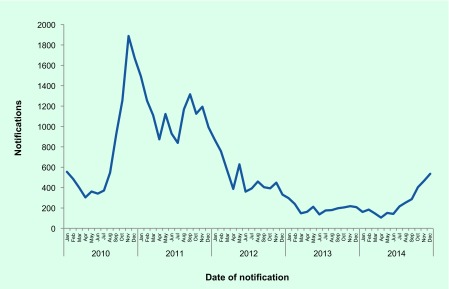
Pertussis notifications by month of onset, NSW, January 2010 to December 2014

There was an upward trend in the number of pertussis notifications from mid-2014 (**Fig. 1**), increasing from 171 notifications in January to 525 in December. The increase was primarily among school-age children, with the highest rates in the 5–9 year age group (134.6 cases per 100 000 population) and the 10–14 year age group (115.2 cases per 100 000 population). A total of 3131 cases were notified in 2014, up from 2342 in 2013. One death was notified in an unvaccinated infant. Females comprised 55% of cases in 2014.

A total of 400 cases occurred in children in the 0–4 year age group. A total of 79 cases occurred in infants aged less than 12 months, of which 18 were unvaccinated and one in which the parent could not recall. Of those who were vaccinated, 54 were fully vaccinated and six partially vaccinated, for age.

High crude notification rates were seen in most LHDs.

### Invasive pneumococcal disease

A total of 517 cases of IPD were notified in 2014, up from 471 in 2013. Females comprised 50% of cases in 2014. A total of 53 deaths were identified, three of which were in infants: one aged 1 month (serotype 22F, non-vaccine type), one aged 2 months (serotype 23B, non-vaccine type) and one aged 1 year (serotype 23B, non-vaccine type). Of the remaining deaths, four were in the 33–49 year age group, 14 in the 50–64 year age group and 32 in people aged 65 years or older. Pneumonia was the leading cause of IPD in adults aged 50 years and over (65%) and bacteraemia in children under the age of 5 years (47%). Of the 390 cases that occurred in the age groups that are followed up by PHUs (0–4 year age group or 50 years or over), 16 (4%) were notified in Aboriginal people, among whom case notification rates were higher than in non-Aboriginal people (26.4 and 12.6 per 100 000, respectively, *P* = 0.010).

The rate of IPD in children under 5 years of age was 13.9 cases per 100 000 population, up from 12.5 cases per 100 000 in 2013. The proportion of cases under 5 years of age with meningitis was 6% higher than in previous years, up from 10% to 16% (*P* = 0.03). Serotype 19A was the leading cause of all IPD in children (26%) followed by 19F (11%), both of which are included in the current 13-valent vaccine. In children under 5 years of age, 51% of disease was caused by non-vaccine serotypes and this rate continues to increase. Vaccination data were available for 100% (69 cases) of notifications under the age of 5 years. A total of 50 cases (73%) were fully vaccinated and 17 cases (24%) were either partially vaccinated or too young to have received their first dose. There were two cases (3%) whose parents chose not to vaccinate. There were 16 cases (26%) of vaccine serotype disease in fully vaccinated children. Serotype 19A accounted for 59% of vaccine failures and serotypes 3 (24%), 19F (12%) and 14 (5%) were responsible for the remainder of cases. The number of vaccine failures in children under 5 years reported in 2014 was higher than previously reported.

### Rubella

Ten cases of rubella were notified in 2014, seven of whom were female. Cases ranged from 7 to 48 years of age. No cases of congenital rubella were notified in 2014.

### Tetanus

One case of tetanus was notified in 2014 in an adult who had not been vaccinated.

## Discussion

The majority of notifiable vaccine-preventable diseases remain well controlled in NSW with case counts and rates well below historical levels. However, both measles and pertussis remain a persistent public health challenge. High immunization rates and rapid public health response are required to maintain measles elimination and control outbreaks of pertussis.

The last notified case of respiratory diphtheria in NSW occurred in 1991. Cutaneous diphtheria does not meet the NSW or national case definition (clinical evidence of pharyngitis/laryngitis or toxic symptoms), but due to the risk of transmission to the respiratory tract, public health follow-up is warranted. The inclusion of cutaneous diphtheria as a notifiable disease in future national surveillance is under consideration.

Measles cases that were acquired outside Australia in 2014 were predominantly imported from the Philippines. The Philippines experienced a measles outbreak associated with increased measles circulation in the Western Pacific Region (*6*) that was exacerbated by the disruption associated with Typhoon Haiyan. The most common genotype observed in NSW was B3, which is the predominant genotype in the Philippines.

Children receive their first dose of measles-containing vaccine at 12 months of age per the National Immunization Program schedule. (*1*) In 2014, there were five cases of measles in children under 12 months, of which three acquired disease outside of Australia in measles-endemic areas (the Philippines and Indonesia). Although these children were too young to be vaccinated under the national schedule, the Australian Immunization Handbook advises that measles–mumps–rubella vaccine can be given as early as 9 months, (*1*) which may be appropriate when infants travel to areas that are endemic or are experiencing an outbreak.

IMD cases continue to decrease following the implementation of the national meningococcal C immunization programme in 2003. (*7*) No serogroup C cases were notified in 2014. Serogroup B remains the most frequent cause of IMD in NSW; however, even in the absence of a publicly funded vaccine, notifications have been decreasing (22 cases notified in 2014, and 27 in 2013). Seven cases of IMD were caused by serogroup W135, which is a slight increase over the preceding years. Further study on this serogroup—particularly further genetic characterization (*8*)—may help elucidate a connection, if any, to the global spread of W135 following the 2000 Haj. (*9*)

The highest rate of IMD (1.2 notifications per 100 000) was observed in the Hunter New England LHD (11 cases), with an unusually high proportion of serogroup Y (four of the 11 cases; 36.4%). This represents more than half of the seven serogroup Y cases in NSW in 2014. No epidemiological link was identified between the cases.

Pertussis notifications increased markedly from mid-way through 2014, indicating the potential beginning of an epidemic. Despite Australia having a long-established vaccination programme for pertussis, periodic epidemics do occur. (*10*) Epidemics of pertussis occurred in 2008–09 and 2010–11 and generally occur every three to four years. Previous pertussis epidemics have been shown to be associated with increased infant hospitalizations and increased morbidity and mortality. (*10*) This is reflected in the 2014 data, with a high proportion of cases in those aged 14 years or less. Notifications will continue to be monitored.

The rate of IPD increased in 2014 across most age groups (except people aged 65–84 years). For the first time since 13-valent pneumococcal conjugate vaccine (PCV-13) was introduced in 2011, the rate of IPD in children under 5 years increased, to 13.9 per 100 000 (up from 12.6 per 100 000 in 2013), although this is still lower than the rate of IPD when PCV-13 was introduced in 2011 (19.0 per 100 000). The proportion of IPD due to non-vaccine serotypes has increased by 29% since PCV-13 introduction. The increase in IPD incidence in children under 5 years is concerning; in addition, 11 cases (16%) were diagnosed with meningitis—a significant increase in life threating illness. This is the highest percentage of meningitis reported in children (average 6% per year) since pneumococcal surveillance began in 1990. Higher rates of IPD in Aboriginal populations were observed despite high vaccination coverage.

Data from NSW disease surveillance systems are subject to the limitations inherent in any disease surveillance programme. The number of notifications reflects health-seeking behaviour and testing practices in NSW. The effect of this limitation will vary by condition. For high-severity diseases such as IMD or measles, it is likely that all cases will be captured in surveillance, but for conditions such as pertussis, notifications will represent only a proportion of the actual cases. In these cases, numbers of notifications will represent trends rather than absolute numbers.

## Conclusion

The majority of vaccine-preventable diseases remain well controlled in NSW. While the number of measles and IPD notifications increased, crude incidence rates remained low. The exception observed was pertussis, which had increasing numbers of notifications, a phenomenon expected every three to four years as immunity from either vaccination or infection is not long-lasting. Control of pertussis in NSW, as elsewhere, remains a challenge, with waning pertussis immunity following vaccination or infection leading to periodic outbreaks.
